# Circulating angiotensin II deteriorates left ventricular function with sympathoexcitation via brain angiotensin II receptor

**DOI:** 10.14814/phy2.12514

**Published:** 2015-08-19

**Authors:** Keisuke Shinohara, Takuya Kishi, Yoshitaka Hirooka, Kenji Sunagawa

**Affiliations:** 1 Departments of Cardiovascular Medicine, Kyushu University Graduate School of Medical SciencesFukuoka, Japan; 2 Department of Advanced Therapeutics for Cardiovascular Diseases, Kyushu University Graduate School of Medical SciencesFukuoka, Japan; 3 Department of Cardiovascular Regulation and Therapeutics, Kyushu University Graduate School of Medical SciencesFukuoka, Japan

**Keywords:** Angiotensin II, heart failure, sympathetic nerve activity

## Abstract

Sympathoexcitation contributes to the progression of heart failure. Activation of brain angiotensin II type 1 receptors (AT_1_R) causes central sympathoexcitation. Thus, we assessed the hypothesis that the increase in circulating angiotensin II comparable to that reported in heart failure model affects cardiac function through the central sympathoexcitation via activating AT_1_R in the brain. In Sprague-Dawley rats, the subcutaneous infusion of angiotensin II for 14 days increased the circulating angiotensin II level comparable to that reported in heart failure model rats after myocardial infarction. In comparison with the control, angiotensin II infusion increased 24 hours urinary norepinephrine excretion, and systolic blood pressure. Angiotensin II infusion hypertrophied left ventricular (LV) without changing chamber dimensions while increased end-diastolic pressure. The LV pressure**–**volume relationship indicated that angiotensin II did not impact on the end-systolic elastance, whereas significantly increased end-diastolic elastance. Chronic intracerebroventricular infusion of AT_1_R blocker, losartan, attenuated these angiotensin II-induced changes. In conclusion, circulating angiotensin II in heart failure is capable of inducing sympathoexcitation via in part AT_1_R in the brain, subsequently leading to LV diastolic dysfunction.

## Introduction

Sympathetic hyperactivity is a cardinal manifestation of heart failure, and contributes to worsening of mortality, left ventricular (LV) remodeling, and dysfunction (Cohn et al. 1984; Ferguson et al. 1990; Triposkiadis et al. 2009; Esler 2010; Malpas 2010; Hirooka et al. 2013; Florea and Cohn 2014). Although the sympathetic activity is modulated and controlled by brain, the mechanism by which brain senses the condition of heart failure from the body and leads to sympathoexcitaion have not been fully understood. Circulating angiotensin II is one of the major inputs from a variety of sources in the body to the brain (Leenen 2007, 2014), and is increased in the heart failure model animals (Schunkert et al. 1993; Leenen et al. 1999a,b; Kang et al. 2008, 2009). In addition, the heart failure patients with increased circulating angiotensin II had higher neurohormonal activation and poor prognosis (Roig et al. 2000). Angiotensin II mediates most of its relevant biological effects through angiotensin II type 1 receptor (AT_1_R) (Timmermans et al. 1993). AT_1_R are found throughout the central nervous system and are highly expressed in areas regulating sympathetic outflow, such as circumventricular organs, hypothalamus, and medulla (McKinley et al. 2003). It has been demonstrated that brain AT_1_R-induced superoxide causes sympathetic hyperactivity (Bains and Ferguson 1995; Ito and Sved 1996; Cato and Toney 2005), however, it remains unknown whether circulating angiotensin II levels observed in heart failure induce central sympathetic activation or not.

In the brain, subfornical organ (SFO) is one of the circumventricular organs lying outside the blood-brain barrier (BBB) and thus responds to the circulating angiotensin II. Angiotensinergic neurons in the SFO are projected to hypothalamic nuclei that modulate sympathetic activity (Johnson and Gross 1993; Bains and Ferguson 1995). Thus, circulating angiotensin II can act on the AT_1_R in the SFO, resulting in sympathoexcitation through the activation of hypothalamic neurons. Rostral ventrolateral medulla (RVLM) in the brainstem is another important cardiovascular control region, located inside the BBB. RVLM is a vasomotor center that contains sympathetic premotor neurons and determines basal sympathetic activity (Dampney 1994; Guyenet 2006). Despite circulating angiotensin II being incapable of crossing the BBB, AT_1_R is highly expressed in the RVLM inside the BBB (McKinley et al. 2003), and the activation of AT_1_R in the RVLM causes symapathoexcitation (Ito and Sved 1996; Allen et al. 2006). However, it has been unclear whether the increased circulating angiotensin II in heart failure induces AT_1_R activation in the RVLM as well as SFO.

Considering these backgrounds, in the present study, we hypothesized that the increase in circulating angiotensin II deteriorates LV function through the central sympathoexcitation via AT_1_R-induced superoxide in the brain. To assess our hypothesis, we considered that the known various mechanisms of sympathoexcitation in myocardial infarction-induced heart failure should be excluded, and that we increased circulating angiotensin II by subcutaneous infusion in normal rats, not heart failure model rats, without the known various mechanisms of sympathoexcitation in myocardial infarction-induced heart failure. We used low doses of angiotensin II resulting in the plasma angiotensin II levels within the pathophysiological range and comparable to that in heart failure model. In the brain, superoxide was also measured by dihydroethidium (DHE) staining because the activation of AT_1_R/NAD (P) H oxidase mainly produces superoxide (Chan et al. 2005; Nozoe et al. 2008; Kishi et al. 2010, 2011).

## Materials and Methods

### Animals and general procedure

This study was reviewed and approved by the Committee on Ethics of Animal Experiments, Kyushu University Graduate School of Medical Sciences, and it was performed according to the Guidelines for Animal Experiments of Kyushu University.

The study was performed using male Sprague-Dawley (SD) rats (SLC Japan, Hamamatsu, Japan) weighing 220–280 g. All rats were housed in a room with controlled lighting, temperature, and humidity and fed standard chow and water ad libitum. The rats assigned to one of four groups: (1) treatment with subcutaneous (SC) vehicle infusion and intracerebroventricular (ICV) vehicle infusion (VEH group); (2) treatment with SC angiotensin II infusion and ICV vehicle infusion (AII group); (3) treatment with SC angiotensin II infusion and ICV losartan (AII + LOS group); and (4) treatment with SC angiotensin II, SC hydralazine, and ICV vehicle (AII + HYD group). The osmotic mini-pumps and ICV cannulae (Alzet, DURECT Corporation, Cupertino, CA) were implanted for SC infusions and ICV infusions. SC angiotensin II (100 ng kg^−1^ min^−1^), ICV losartan (1 mg kg^−1^ day^−1^), and SC hydralazine (8 mg kg^−1^ day^−1^) were infused for 2 weeks. In a preliminary experiment, we confirmed that 100 ng kg^−1^ min^−1^ SC angiotensin II infusion for 2 weeks increased plasma angiotensin II levels comparable to those in heart failure model rats, which was consistent with previous studies (Leenen et al. 1999a,b; Kang et al. 2008, 2009; Huang et al. 2010; ). The doses of losartan and hydralazine were determined according to previous studies (Zhang et al. 1999; Kang et al. 2008; Huang et al. 2009; Nishihara et al. 2012a,b). The systemic and peripheral infusion of losartan at the present dose has already been reported not to alter hemodynamics (Zhang et al. 1999).

### Measurements of plasma angiotensin II levels, urinary norepinephrine excretion, hexamethonium-induced blood pressure changes, and blood pressure

Blood was collected at the end of the 2 weeks treatment period. Plasma angiotensin II levels were measured by double antibody radioimmunoassay. Urine was collected from each rat for 24 h in a metabolic cage before and after treatment. Urinary norepinephrine concentration was measured by using high-performance liquid chromatography to calculate urinary norepinephrine excretion as a marker of sympathetic activity, as described in our previous studies (Nozoe et al. 2008; Kishi et al. 2010, 2011). As another indicator of sympathetic activity, the maximal decrease in the mean arterial pressure (MAP) induced by intravenous injection of hexamethonium hydrochloride, a ganglionic blocker, was also measured (Koga et al. 2008). The femoral artery and vein were cannulated under the anesthesia with pentobarbital sodium (50 mg kg^−1^, intraperitoneal injection) to measure the MAP and inject the drug at the end of the 2 weeks treatment. Systolic blood pressure (SBP) was measured by using the tail–cuff method (BP-98A; Softron, Tokyo, Japan) in each group every 2 days before and during the treatment.

### Echocardiographic and hemodynamic studies

LV end-diastolic diameter (LVDd), LV end-systolic diameter (LVDs), and LV wall thickness were measured, and LV percentage fractional shortening (%FS) were determined by echocardiography under light anesthesia with an intraperitoneal injection of pentobarbital sodium (30 mg kg^−1^). Echocardiography was performed after the 2 weeks treatment using an Aloka Prosound SSD 5000 (Tokyo, Japan) with a 7.5-MHz imaging linear scan probe transducer, as described previously (Ito et al. 2012; Honda et al. 2013; Ogawa et al. 2013; Kishi et al. 2014). Hemodynamic study was performed at the end of the 2 weeks treatment period. The rats were anesthetized with pentobarbital sodium (50 mg kg^−1^, intraperitoneal injection) and artificially ventilated (70 breaths min^−1^ with 95% O2). Body temperature was maintained at 37.0°C with a controlled heating pad. A microtip pressure**–**volume catheter (1.9 Fr; Scisence, London, ON, Canada) was inserted into the right carotid artery and advanced to the LV. Signals were continuously monitored at a sampling rate 1000 Hz using an Advantage pressure**–**volume measurement system (Scisence Inc.). We evaluated LV end-diastolic pressure (EDP), maximum rate of pressure increase (+dP/dt max), maximum rate of pressure reduction (−dP/dt max), and LV end-diastolic pressure**–**volume relation slope (EDPVR) (Pacher et al. 2008).

### Measurement of organ weight and histological analysis

After 2 weeks treatment, the rats were killed and their hearts and lungs were harvested. The ventricles and lungs were rinsed with isotonic saline (0.9% NaCl), then dissected and weighed. The weights of the total heart and lung were normalized to the body weight and used as an index of ventricular hypertrophy and pulmonary congestion respectively (Honda et al. 2013). The LV was fixed in 10% formalin and embedded in paraffin. The tissue was sectioned into 5 *μ*m slices, and stained with hematoxylin-eosin or Masson trichrome for evaluation of cardiomyocyte hypertrophy or interstitial fibrosis. To determine the cardiomyocyte size, the cross-sectional area of myocardial fibers was measured at the level of nuclei in at least 100 cardiomyocytes per heart using a 40 times magnified lens. The area of interstitial fibrosis was calculated as the ratio of the sum of the total area of interstitial fibrosis to the sum of the total connective tissue area and the cardiomyocyte area in all the LV fields of the section. Three sections were examined per heart using a four times magnified lens for the fibrosis analysis (Honda et al. 2013). A calibrated digital camera (Model BZ-9000, Keyence Co., Osaka, Japan) and an imaging analysis program (BZH1C; Keyence Co., Tokyo, Japan) were used in the histological analysis.

### Dihydroethidium staining

Brain superoxide anion levels were estimated by DHE staining based on previous studies (Kimura et al. 2005; Nishihara et al. 2012a,b). After 2 weeks treatment, brains were removed, quickly frozen, and unfixed frozen regions of the SFO or RVLM were cut into 25-*μ*m sections using a cryostat and transferred to glass slides. Sections were thawed at room temperature, rehydrated with PBS, and incubated for 10 min in the dark with the superoxide-specific fluorogenic probe DHE (1 *μ*mol L^−1^) at 37°C. After washing with PBS, DHE fluorescence was visualized by confocal microscopy using an excitation wavelength of 543 nm and a rhodamine emission filter.

### Statistical analysis

All values are expressed as the mean ± SEM. Two-way repeated measures anova with Bonferroni post hoc tests was used to compare SBP between groups. One-way anova with Bonferroni post hoc tests was used to compare all other parameters between groups. Differences were considered to be statistically significant for *P* < 0.05.

## Results

### Plasma angiotensin II levels, urinary norepinephrine excretion, and hexamethonium-induced MAP reduction

**Figure 1:**
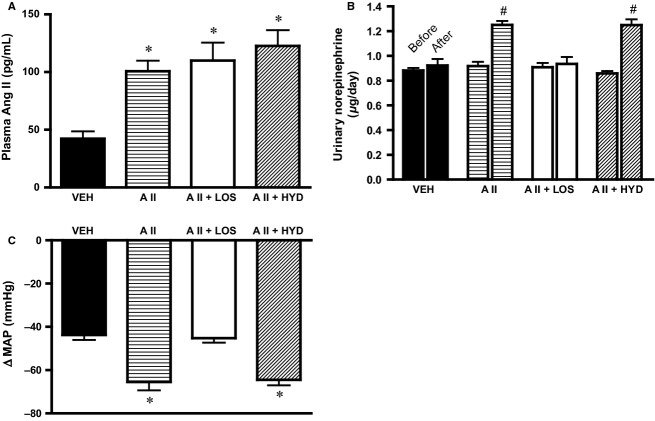
(A) Plasma angiotensin II levels, (B) urinary norepinephrine excretion, and (C) hexamethonium-induced mean arterial pressure (MAP) reduction, in rats treated with subcutaneous vehicle and intracerebroventricular infusion of vehicle (VEH), treated with subcutaneous angiotensin II and intracerebroventricular infusion of vehicle (AII), treated with subcutaneous angiotensin II and intracerebroventricular infusion of losartan (AII + LOS), or treated with subcutaneous angiotensin II, subcutaneous hydralazine, and intracerebroventricular infusion of vehicle (AII + HYD) (A, *n* = 8 in each; B, *n *= 8 in VEH, 12 in AII, 8 in AII + LOS, and 8 in AII + HYD; C, *n *= 4 in each). Urinary norepinephrine excretion and hexamethonium-induced MAP reduction are parameters of sympathetic activity. The data of plasma angiotensin II levels and hexamethonium-induced MAP reduction was obtained from the rats after treatment; the data of urinary norepinephrine excretion was obtained from the rats before and after treatment. *P < 0.05 versus VEH; ^#^*P *< 0.05 versus before in each group. MAP, mean arterial pressure.

Plasma angiotensin II levels were increased approximately 2- to 2.5-fold in A II, AII + LOS, and AII + HYD groups compared with VEH group (Fig.1A). These increased plasma angiotensin II levels were within the pathophysiological range and comparable to those in chronic heart failure model (Leenen et al. 1999a,b; Kang et al. 2009; Kleiber et al. 2010). Urinary norepinephrine excretion was significantly increased in AII group compared with VEH group. Angiotensin II-induced increase in urinary norepinephrine excretion was inhibited in AII + LOS group, but not in AII + HYD group (Fig.1B). The decrease in MAP induced by hexamethonium was significantly greater in AII and AII + HYD group compared with VEH group. The decrease in MAP induced by hexamethonium in AII + LOS group was similar to that in VEH group (Fig.1C). These data of urinary norepinephrine excretion and hexamethonium-induced MAP reduction suggest that the sympathetic activity was significantly increased in AII and AII + HYD group, not in AII + LOS group, compared with VEH group.

### Blood pressure

**Figure 2:**
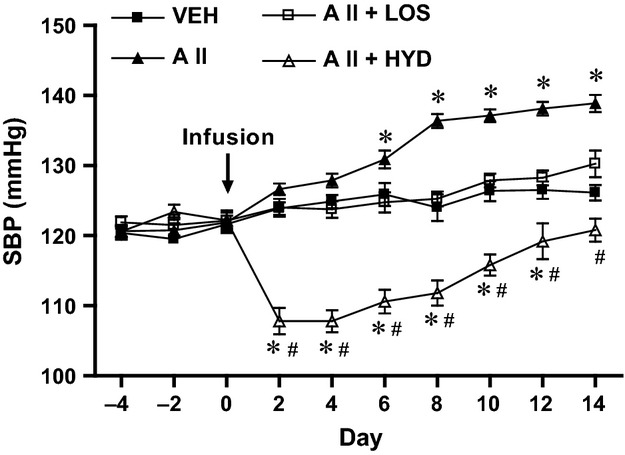
Time courses of systolic blood pressure (SBP) in rats treated with subcutaneous vehicle and ICV vehicle (VEH), treated with subcutaneous angiotensin II and intracerebroventricular infusion of vehicle (AII), treated with subcutaneous angiotensin II and intracerebroventricular infusion of losartan (AII + LOS), or treated with subcutaneous angiotensin II, subcutaneous hydralazine, and intracerebroventricular infusion of vehicle (AII + HYD) (*n *= 8 in VEH, 8 in AII, 8 in AII + LOS, and 5 in AII + HYD). *P < 0.05 versus VEH; ^#^*P *< 0.05 versus AII + LOS in AII + HYD.

Chronic infusion of low doses of angiotensin II resulted in a gradually developing pressor response (Fig.2), indicating a good model with sympathetic hyperactivity (Simon et al. 1995; Li et al. 1996). The ICV losartan inhibited the angiotensin II-induced pressor response in AII + LOS group. In AII + HYD group, the decreased SBP was observed from the early treatment period, and the SBP was lower than that of AII + LOS group at the end of 2 weeks treatment.

### Echocardiography

LVDd, LVDs, and %FS were not significantly different among the groups (Table1). However, the LV wall thickness (interventricular septum thickness plus LV posterior wall thickness) was significantly increased in AII and AII + HYD group compared with VEH group (Table1). Angiotensin II-induced increase in LV wall thickness was inhibited in AII + LOS group.

**Table 1:** Echocardiographic data

	VEH	AII	AII + LOS	AII + HYD
LVDd (mm)	6.08 ± 0.09	6.11 ± 0.08	6.25 ± 0.06	6.34 ± 0.08
LVDs (mm)	2.78 ± 0.06	2.90 ± 0.05	2.84 ± 0.05	2.88 ± 0.05
IVS + PW (mm)	3.23 ± 0.04	3.63 ± 0.08*	3.39 ± 0.04	3.52 ± 0.09*
%FS, (%)	54.4 ± 0.4	52.6 ± 0.5	54.6 ± 0.8	54.6 ± 1.0

Values are means ± SE. Echocardiographic data in rats treated with subcutaneous vehicle and intracerebroventricular infusion of vehicle (VEH), treated with subcutaneous angiotensin II and intracerebroventricular infusion of vehicle (AII), treated with subcutaneous angiotensin II and intracerebroventricular infusion of losartan (AII + LOS), or treated with subcutaneous angiotensin II, subcutaneous hydralazine, and intracerebroventricular infusion of vehicle (AII + HYD) (*n *= 8 in VEH, 8 in A II, 8 in A II + LOS, and 5 in A II + HYD). LVDd, left ventricular end-diastolic diameter; LVDs, left ventricular end-systolic diameter; IVS + PW, interventricular septum thickness plus posterior wall thickness; %FS, percentage fractional shortening.

* *P *< 0.05 versus VEH.

### Hemodynamic study

**Figure 3:**
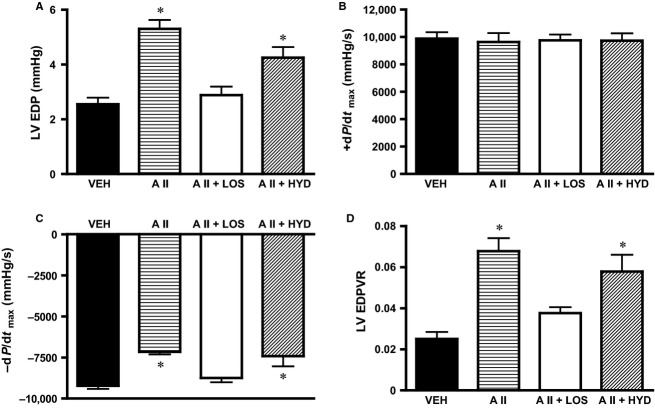
(A) Left ventricular (LV) end-diastolic pressure (LVEDP), (B) maximum rate of pressure increase (+dP/dt max), (C) maximum rate of pressure reduction (-dP/dt max), (D) LV end-diastolic pressure–volume relation slope (EDPVR). Hemodynamic data in rats treated with subcutaneous vehicle and intracerebroventricular infusion of vehicle (VEH), treated with subcutaneous angiotensin II and intracerebroventricular infusion of vehicle (AII), treated with subcutaneous angiotensin II and intracerebroventricular infusion of losartan (AII + LOS), or treated with subcutaneous angiotensin II, subcutaneous hydralazine, and intracerebroventricular infusion of vehicle (AII + HYD) (*n *= 5 in each). *P < 0.05 versus VEH.

LVEDP was increased in AII and AII + HYD group, and was not changed in AII + LOS group, compared with VEH group (Fig.3A). Although +dP/dt max were not changed among groups (Fig.3B), −dP/dt max was decreased in AII and AII + HYD group, and was not changed in AII + LOS group, compared with VEH group (Fig.3C). LV EDPVR was increased in AII and AII + HYD group, and was not changed in AII + LOS group, compared with VEH group (Fig.3D).

### Organ weight and histological analysis

**Figure 4:**
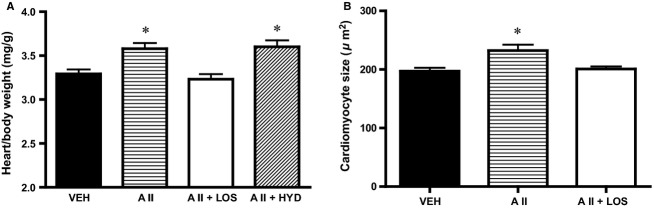
(A) Heart/body weight by measurement of organ weight in rats treated with subcutaneous vehicle and intracerebroventricular infusion of vehicle (VEH), treated with subcutaneous angiotensin II and vehicle (AII), treated with subcutaneous angiotensin II and intracerebroventricular infusion of losartan (AII + LOS), or treated with subcutaneous angiotensin II, subcutaneous hydralazine, and intracerebroventricular infusion of vehicle (AII + HYD) (*n *= 8 in VEH, 8 in AII, 8 in AII + LOS, and 5 in AII + HYD). (B) Left ventricular cardiomyocyte size by histological analysis in VEH, AII, and A II + LOS (*n *= 5 in each). *P < 0.05 versus VEH.

Heart/body weight was significantly increased in AII and AII + HYD group, but was not changed in AII + LOS group, compared with VEH group (Fig.4A). The lung/body weight was not different among groups (VEH, 4.30 ± 0.05 mg g^−1^; AII, 4.36 ± 0.07 mg g^−1^; AII + LOS, 4.42 ± 0.06 mg g^−1^; AII + HYD, 4.38 ± 0.06 mg g^−1^; *n* = 8 in VEH, 8 in AII, 8 in AII + LOS, and 5 in AII + HYD; ns). Histological analysis was performed in VEH, AII, and AII + LOS groups, and showed that the cardiomyocyte size was greater in AII group than that in VEH group. The ICV losartan inhibited angiotensin II-induced cardiomyocyte hypertrophy (Fig.4B). The degree of myocardial interstitial fibrosis did not differ significantly among the groups (VEH, 0.86 ± 0.10%; AII, 0.95 ± 0.05%; AII + LOS, 0.87 ± 0.05%; *n* = 5 in each; ns).

### Superoxide production

**Figure 5:**
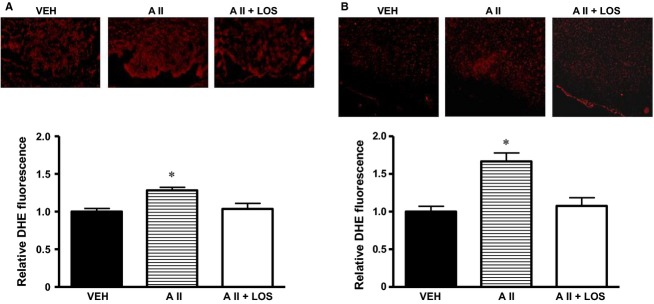
Representative images showing dihydroethidium (DHE) fluorescence and summary of DHE fluorescence intensity in (A) subfornical organ and (B) rostral ventrolateral medulla of rats treated with subcutaneous vehicle and intracerebroventricular infusion of vehicle (VEH), treated with subcutaneous angiotensin II and intracerebroventricular infusion of vehicle (A II), or treated with subcutaneous angiotensin II and intracerebroventricular infusion of losartan (A II + LOS) (*n *= 4 in each). Data are expressed relative to VEH. *P < 0.05 versus VEH.

Dihydroethidium staining was performed in VEH, II, and A II + LOS groups. The DHE fluorescence levels were slightly but significantly higher in the SFO of AII group than that of VEH group. This increased DHE fluorescence levels in the SFO were inhibited in AII + LOS group (Fig.5A). In the RVLM, the DHE fluorescence levels were significantly increased in AII group compared with VEH group, and the angiotensin II-induced increase in the DHE fluorescence levels was inhibited in AII + LOS group (Fig.5B).

## Discussion

The main findings from this study are that the increase in the circulating angiotensin II induced LV hypertrophy and LVEDP elevation through central sympathoexcitation via in part AT_1_R in the brain. It is widely known and recognized that angiotensin II in the brain increases sympathoexcitation, and that increased sympathoexcitation contributes to heart failure. The present new and novel finding is that increased circulating angiotensin II at similar level of myocardial infarction could directory affect brain and cause excessive sympathoexcitation in normal rats without various mechanisms of sympathoexcitation (hemodynamic and cardiopulmonary reflex, baroreflex failure, and/or excess excitation of brain renin-angiotensin system). We would like to emphasis that excess circulating angiotensin II would be one of the major sympathoexciting factors via central renin-angiotensin system in myocardial infarction-induced heart failure.

The increased plasma angiotensin II levels within the pathophysiological range and comparable to those in heart failure models (Leenen et al. 1999a,b; Kang et al. 2009; Kleiber et al. 2010) are induced by the low doses of SC angiotensin II infusion, as demonstrated in the present study. Despite these results, the models treated with the higher doses, such as greater than 400 ng kg^−1^ min^−1^, of SC angiotensin II have been commonly studied to investigate LV remodeling and dysfunction. It is notable that the increased circulating angiotensin II comparable to that in heart failure caused the sympathoexcitation, LV hypertrophic remodeling and diastolic dysfunction. Interestingly, the inhibition of brain AT_1_R by ICV losartan treatment significantly attenuated these angiotensin II-induced LV changes through sympathoinhibition. Although the ICV losartan inhibited the angiotensin II-induced increase in blood pressure as well as LV changes, we did not consider that the inhibition of the LV changes was due to just only blood pressure reduction, because the treatment with hydralazine did not inhibit the angiotensin II-induced LV changes, despite the fact that hydralazine reduced blood pressure to a level lower than that observed in the angiotensin II-infused rats treated with ICV losartan. The results with hydralazine predominantly provided the indication that circulating angiotensin II causes sympathoexcitation and worsen heart failure via brain renin-angiotensin system independent of blood pressure. Moreover, though we did not examine in the present study, our previous study suggested that hydralazine induced sympathoexcitation with the production of oxidative stress in the brain (Kishi et al. 2012; Hirooka et al. 2013). Our recent studies also demonstrated that sympathoinhibition attenuated the LV dysfunction independently of blood pressure reduction in rats with hypertensive heart failure (Honda et al. 2013; Kishi et al. 2014). These findings in the present study are supported by the previous studies that indicated the inhibition of LV dysfunction by ICV losartan in the heart failure models after myocardial infarction (Leenen et al. 1999a,b; Kang et al. 2008), and further suggest that the increased circulating angiotensin II induced by heart failure causes central sympathoexcitaion, leading to LV remodeling and diastolic dysfunction.

Many previous studies have focused either forebrain or brainstem, however, we focused on both SFO in the forebrain and RVLM in the brainstem because the SFO and RVLM are the important regions as a primary sensor for circulating angiotensin II and the cardiovascular center determining sympathetic activity respectively. The RVLM is protected from circulating angiotensin II by BBB, whereas the SFO is influenced directly by circulating angiotensin II. Interestingly, the AT_1_R expression was increased not only in the SFO, but also in the RVLM in the angiotensin II-infused rats in the present study. Angiotensin II is locally synthesized in the brain including the RVLM because of the intrinsic brain renin-angiotensin system (McKinley et al. 2003), and AT_1_R signaling is mediated by a positive feedback of AT_1_R on the transcriptional regulation of the protein in the brain (Zucker et al. 2014). It has also been demonstrated that angiotensinergic sympathoexcitatory pathways project from the SFO to the paraventricular nucleus (PVN) in the hypothalamus, and from the PVN project to the RVLM (Wright et al. 1993; Tagawa and Dampney 1999; Cato and Toney 2005), probably using angiotensin II as a neurotransmitter to enhance the excitability of the postsynaptic neuron in the brain (Ferguson 2009). Together with these previous studies, although we did not assess the components of brain renin-angiotensin system, our findings would suggest that the increased circulating angiotensin II activated AT_1_R in the brain, in part SFO and/or RVLM. In fact, the up-regulation of AT_1_R in the SFO (Wei et al. 2008) and RVLM (Gao et al. 2008), respectively, has been demonstrated in the heart failure model rats. Furthermore, we showed that the superoxide production was increased in the SFO and RVLM in the angiotensin II-infused rats. The superoxide is mainly generated by the activation of AT_1_R and plays a major role in the angiotensin II-induced sympathoexcitaion (Gao et al. 2004; Campese et al. 2005; Han et al. 2005; Sun et al. 2005; Lob et al. 2010; Kishi et al. 2012). It has been demonstrated that an elevated level of superoxide in the SFO and RVLM contributes to the pathogenesis of heart failure (Lindley et al. 2009; Gao et al. 2010; Kishi et al. 2014). The finding that the ICV losartan abolished the increase in superoxide production in the brain in angiotensin II-infused rats further suggests that the increased circulating angiotensin II induced by heart failure was crucially involved in the sympathoexcitaion probably through the increase in superoxide production via in part AT_1_R in the brain. Probably ICV losartan should block AT_1_R in PVN, and similar results with SFO and RVLM would be obtained. However, PVN should be focused in the next experiment.

Our results demonstrated that the increased circulating angiotensin II led to the LV hypertrophic remodeling and diastolic dysfunction through sympathoexcitaion. Interestingly, in the present study, around twofold increased circulating angiotensin II (similar to myocardial infarction-induced heart failure) was not a typical heart failure and was similar to hypertensive heart failure with mild alteration of cardiac function. The LV hypertrophic remodeling was indicated by the increase in heart/body weight, LV wall thickness, and LV cardiomyocyte size in the angiotensin II-infused rats. LV hypertrophic remodeling is involved in diastolic dysfunction, in particular, the increased stiffness and the impaired relaxation (Schraeger et al. 1994; Gaasch and Zile 2004; van. Heerebeek et al. 2006), which we demonstrated by measuring the LV EDPVR and −dP/dt max. In addition to the LV hypertrophy, myocardial fibrosis is considered to induce the LV diastolic dysfunction (Gaasch and Zile 2004). Although the myocardial interstitial fibrosis, unlike the cardiomyocyte size, was not significantly increased in the angiotensin II-infused rats with sympathetic hyperactivity, our findings are also supported by a previous study demonstrating that LV hypertrophy, not fibrosis, is closely related to the increased LV stiffness (Schraeger et al. 1994). Excessive sympathetic outflow overstimulates cardiac *β*1-adrenergic receptors, resulting in cardiomyocyte hypertrophy (Engelhardt et al. 1999) and Ca^2+^ leaking from the sarcoplasmic reticulum (Marx et al. 2000) and subsequently increasing LV stiffness and impairing LV relaxation. Therefore, sympathoinhibition induced by the ICV losartan probably protected *β*1-adrenergic receptors in the heart from catecholamine stimulation. Moreover, we should focus on the T cell activation and chronic inflammation induced by sympathoexcitation should also modulate *β*-adrenergic receptors. In the present experiments, we did not assess the inflammation. In future studies, these aspects must be clarified.

We focused on the increased circulating angiotensin II resulting from heart failure as an important input from the body for leading to central sympathoexcitaion that contributes to the progression of heart failure. Heart failure can induce a variety of sympathoexcitatory inputs to the brain, such as the increased circulating aldosterone and cytokines, and the activation of the peripheral chemoreceptors, as well as the increased circulating angiotensin II (Leenen 2007). Therefore, we used the angiotensin II-infused model, not the heart failure model such as myocardial infarction model, to elucidate the role of the increased circulating angiotensin II in sympathoexcitaion. The increased circulating angiotensin II by the low doses (100 ng kg^−1^ min^−1^) of SC angiotensin II infusion for 2 weeks led to the LV hypertrophy and diastolic dysfunction, but not the LV fibrosis, through sympathoexcitaion. Both LV hypertrophy and fibrosis induced by the same dose of SC angiotensin II for 4 weeks was previously demonstrated, although neither sympathetic activity nor LV function were investigated (Grobe et al. 2007). It has also been demonstrated that the chronic sympathetic hyperactivity for a longer period, more than 6 weeks, can increase the LV fibrosis in hypertension or hypertensive heart failure model (Levick et al. 2010; Honda et al. 2013;). Taken together, the rats infused with the low doses of angiotensin II for a longer period might be used a model with the chronic sympathetic hyperactivity leading to advanced LV remodeling and dysfunction. We demonstrated that ICV losartan attenuated the angiotensin II-induced LV remodeling and dysfunction through sympathoinhibition. The possibility of the direct peripheral actions of the ICV losartan was excluded by the observation that the same doses of SC losartan had no effect (plasma angiotensin II levels, 95.3 ± 13.9 pg mL^−1^, *n* = 8; urinary norepinephrine excretion, 1.23 ± 0.05 *μ*g day, *n* = 8; LV wall thickness, 3.54 ± 0.11 mm, *n* = 5; LVEDP, 3.99 ± 0.16 mmHg, *n* = 5: all these parameters were not significantly different from those of AII group).

There are several study limitations. First, we used subcutaneous angiotensin II infusion in only single dose to increase circulating angiotensin II instead of using models with heart failure, because known various mechanisms of sympathoexcitation in myocardial infarction-induced heart failure should be excluded. However, we also recognize that further experiments using other ideal models with heart failure and/or with lower dose angiotensin II treatment should be done. Second, the results of ICV losartan could not be valid, and we also consider that site-specific knockout or deletion of AT_1_R should be done. Third, our results of DHE staining were not sufficient, and only these results could not show the effects of ICV losartan on superoxide production in SFO and RVLM. However, in our previous studies, ICV losartan reduced oxidative stress measured by other methods in the bilateral RVLM (Hirooka et al. 2013). We consider that ICV losartan could reduce superoxide in the SFO and RVLM. Third, we provided the indirect markers of sympathoexcitation, and did not do the direct measurements of sympathoexcitation. Because we could not do the conscious and long-term (for 14 days) renal sympathetic nerve recording in rats, we evaluated sympathoexcitation by urinary norepinephrine excretion as previously used (Kishi et al. 2004, 2010, 2011, 2012, 2014; Kimura et al. 2005; Koga et al. 2008; Nozoe et al. 2008; Ogawa et al. 2013). However, conscious direct nerve recording will strength our results.

## Conclusion

In conclusion, increased circulating angiotensin II in heart failure is an important input for central sympathoexcitaion, leading to LV remodeling and diastolic dysfunction. In addition, superoxide production in the brain might be involved in the circulating angiotensin II-induced sympathoexcitaion. The increased circulating angiotensin II, sympathoexcitaion, LV remodeling, and dysfunction may play a major role in the vicious cycle of heart failure.
